# Developing a Polygenic Risk Score with Age and Sex to Identify High-Risk Myopia in Taiwan

**DOI:** 10.3390/biomedicines12071619

**Published:** 2024-07-20

**Authors:** Hui-Ju Lin, Yu-Te Huang, Wen-Ling Liao, Yu-Chuen Huang, Ya-Wen Chang, Angel L. Weng, Fuu-Jen Tsai

**Affiliations:** 1 Department of Ophthalmology, China Medical University Hospital, Taichung 404327, Taiwan; 002396@tool.caaumed.org.tw (H.-J.L.); tonyhuang791112@gmail.com (Y.-T.H.); 2 School of Chinese Medicine, China Medical University, Taichung 404328, Taiwan; yuchuen@mail.cmu.edu.tw; 3 Center for Personalized Medicine, China Medical University Hospital, Taichung 404327, Taiwan; wl0129@mail.cmu.edu.tw; 4 Graduate Institute of Integrated Medicine, China Medical University, Taichung 404328, Taiwan; 5 Genetic Center, Department of Medical Research, China Medical University Hospital, Taichung 404327, Taiwan; windy87518@gmail.com; 6 American School in Taichung, Taichung 406051, Taiwan; angellweng96@gmail.com; 7 Department of Medical Genetics, China Medical University Hospital, Taichung 404327, Taiwan; 8 Children’s Hospital of China Medical University, Taichung 404327, Taiwan

**Keywords:** genome-wide association study (GWAS), polygenic risk score (PRS), myopia, spherical equivalent, iHi data platform

## Abstract

Myopia is the leading cause of impaired vision, and its prevalence is increasing among Asian populations. This study aimed to develop a polygenic risk score (PRS) followed by replication to predict myopia in the Taiwanese population. In total, 23,688 participants with cycloplegic autorefraction-measured mean spherical equivalent (SE), genetic, and demographic data were included. The myopia PRS was generated based on genome-wide association study (GWAS) outcomes in a Taiwanese population and previously published GWAS reports. The results demonstrated that the inclusion of age and sex in the PRS had an area under the curve (AUC) of 0.80, 0.78, and 0.73 (*p* < 0.001) for participants aged >18 years with high (SE < −6.0 diopters (D); n = 1089), moderate (−6.0 D < SE ≤ −3.0 D; n = 3929), and mild myopia (−3.0 D < SE ≤ −1.0 D; n = 2241), respectively. Participants in the top PRS quartile had a 1.30-fold greater risk of high myopia (95% confidence interval = 1.09–1.55, *p* = 0.003) compared with that in the remaining participants. Further, a higher PRS significantly increased the risk of high myopia (SE ≤ −2.0 D) in children ≤6 years of age (*p* = 0.027). In conclusion, including the PRS, age, and sex improved the prediction of high myopia risk in the Taiwanese population.

## 1. Introduction

Myopia has emerged as a significant public health concern worldwide because of its association with vision-threatening complications [1,2,3]. The incidence of complications, including retinal detachment, glaucoma, cataracts, and macular degeneration, increases with the severity of myopia. Furthermore, the prevalence of myopia has substantially increased worldwide. A study projected that by 2050, approximately 5 billion individuals will be affected by myopia, with 1 billion experiencing high myopia (defined as a spherical equivalent [SE] of −5.0 D or less) [4].

Myopia is prevalent in Asian countries. For example, a survey conducted in Taiwan revealed that myopia affects 84% of 15–18-year-old students. Among these, 24% were classified as highly myopic [5]. Given that early intervention to prevent myopia development in children can potentially slow its progression, identifying individuals who are more susceptible to myopia and providing them with comprehensive eye care is crucial [6,7,8]. By recognizing the significance of early detection, intervention, and ongoing management of myopia, we can effectively reduce its prevalence and minimize its impact on individuals and communities. This approach will help preserve vision and enhance the quality of life for future generations.

Myopia is widely recognized as a complex trait influenced by a combination of environmental risk factors, lifestyle choices, and genetic variations [9,10,11]. Two environmental and lifestyle factors have been identified as significant contributors to myopia development: long durations of near work and decreased time spent outdoors [7]. In addition to the key role of environmental factors, genetic variations contribute to the development of myopia. Certain genetic factors make individuals more susceptible to myopia, and studies have identified specific genes associated with its occurrence. The interplay between genetic and environmental factors in myopia is an active area of research that aims to elucidate the complex mechanisms underlying this condition.

Advances in genetic technology have linked many loci to myopia through large-scale genome-wide association studies (GWASs) [12]. One GWAS-based meta-analysis identified 336 novel genetic loci, and the sum of the effects explained 18.4% of the heritability and improved the accuracy of myopia prediction [12]. A more practical way to determine individual disease risk is through the polygenic risk score (PRS), which summarizes genetic effects and presents the overall risk of individual genetic susceptibility to a disease [13]. Several studies have examined the role of PRS in myopia progression; however, most of these studies were performed on individuals of European ancestry [14,15]. One PRS model developed in Singaporean Chinese children focused on high and moderate myopia groups. The model with PRS showed an area under the receiver operating characteristic curve (AUC) of 0.64 (95% confidence interval [CI] = 0.57–0.71) for high myopia and 0.57 (95% CI = 0.49–0.64) for moderate myopia [16]. Several strategies can be applied to enhance predictability, including adjusting the number of single-nucleotide polymorphisms (SNPs), geographic data, and physical measurements [17].

In this study, we conducted a large-scale investigation to develop a new East Asian PRS that integrates other demographic data to predict myopia risk in the Taiwanese population. We aimed to narrow the gap in underrepresented populations relative to European individuals and provide a more accurate prediction model for children at high risk of myopia.

## 2. Materials and Methods

### 2.1. Study Population

A total of 23,688 unrelated Taiwanese participants (20,678, 2060, and 950 participants aged >18, 7–18, and ≤6 years, respectively) provided informed consent and were included in the analysis. All Taiwanese participants were of Han Chinese origin and were recruited from China Medical University Hospital (CMUH), Taichung, Taiwan. Refraction was determined using the cycloplegic autorefraction-measured mean spherical equivalent (SE) and was calculated as a sphere plus a half cylinder. The inclusion criteria of participants were SE ≤ −6.0 D in at least one eye for the high myopia group [18], −6.0 D < SE ≤ −3.0 D for the moderate myopia group, and −3.0 D < SE ≤ −1.0 D for the mild myopia group at ages >6 years, and SE ≤ −2.0 D in at least one eye was defined as high myopia at ages ≤ 6 years. Individuals with SE > −1.0 D at ages >6 years and SE > 0.0 D at ages ≤6 years in both eyes were defined as non-myopic [19]. The exclusion criteria were participants with genetic diseases associated with myopia, including Marfan syndrome, Stickler syndrome, or Weill–Marchesani syndrome, as well as participants with glaucoma, cataracts, homocystinuria, and eyeball injury repair. This study was approved by the Institutional Review Board of CMUH (CMUH109-REC2-185).

### 2.2. Data Source

Information on the study population was collected from the iHi data platform, which contains electronic medical records and genetic data established by the Big Data Center of CMUH [20]. Furthermore, an efficient GWAS analytical platform with high-quality controls that can provide real-time GWAS data using detailed phenotypic data from electronic medical records was implemented [20,21,22].

### 2.3. Genotyping and Discovery GWAS

**Figure 1:**
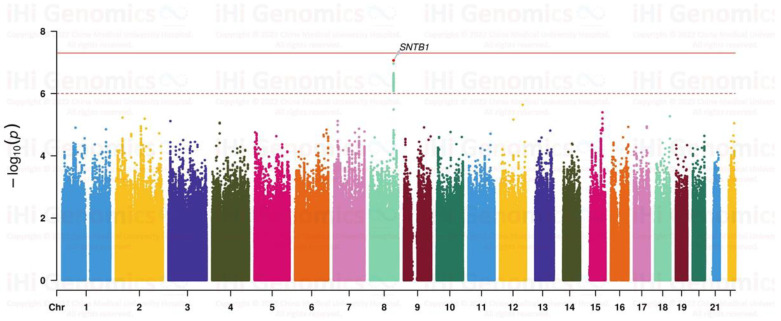
Manhattan plot of the GWAS in the discovery cohort, which included 2541 individuals with high myopia and 7615 individuals with no myopia. The solid line indicates the genome-wide significance threshold of *p* < 5.0 × 10^−8^, while the dashed line indicates the suggestive significance threshold of *p* < 1.0 × 10^−6^. Different colors represented each chromosome.

**Figure 2:**
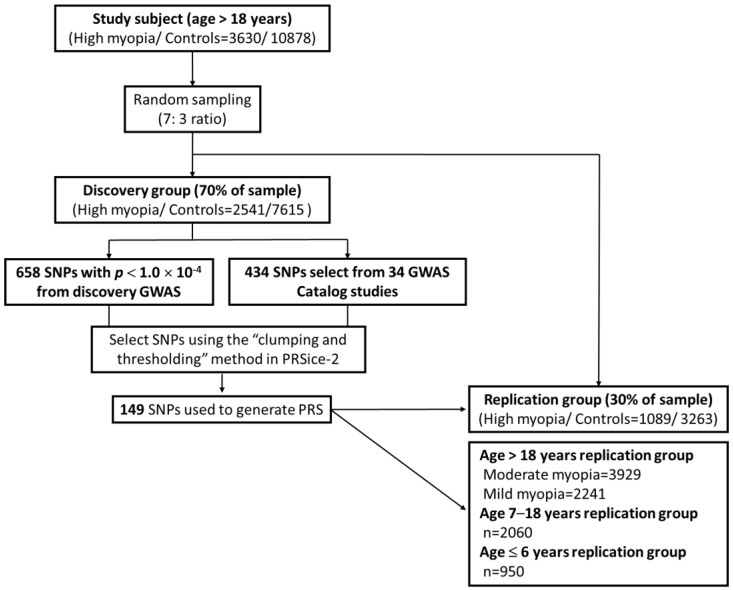
Flowchart of the study design.

Genotyping was performed using the Axiom Taiwan Precision Medicine v1 custom SNP array (Thermo Fisher Scientific, Waltham, MA, USA), which contained approximately 740 K SNPs across the entire human genome. Information on the genotyping, quality control, and imputation methods for all study participants has been described previously [23,24,25,26]. Study participants aged >18 years with high myopia (SE < −6.0 D) and no myopia (SE > −1.0 D) were randomly divided at a ratio of 7:3 into a cohort of 10,156 participants (2541 individuals with high myopia and 7615 control individuals without myopia) for the discovery GWAS (Figure 1). The GWAS results were adjusted for sex and the first six genome-wide principal components (PCs). The remaining 30% of the cohort of 4352 participants (1089 and 3263 individuals with high and no myopia, respectively) were used for replication. Moreover, 2241 and 3929 participants aged >18 years with mild and moderate myopia, respectively, and two independent high-myopia cohorts of participants aged 7–18 years and ≤6 years, respectively, were used for further replication. The detailed study design is shown in Figure 2. In total, 658 SNPs with *p* < 1.0 × 10^−4^ from the GWAS were identified.

### 2.4. PRS Construction

For PRS construction, 658 SNPs with *p* < 1.0 × 10^−4^ from the discovery GWAS and 434 SNPs associated with myopia from 34 GWAS Catalog studies [27] (Supplementary Table S1) were selected using the clumping and thresholding (C + T) method in PRSice–2 v2.3.5 [28]. This algorithm iteratively selects a set of SNPs to form clumps around the index SNP. Based on a pairwise threshold of r^2^ = 0.2, each clump consists of SNPs located within 250 kb of the index SNP and in linkage disequilibrium with the index SNP. After the steps of LD clumping and *p*-value thresholding, 149 SNPs (Supplementary Table S2) and their corresponding estimated β-coefficients of their effect alleles were included as weights in the PRS calculation using PLINK v2.0 [29].

### 2.5. Statistical Analysis

Student’s *t*-test for continuous variables and a chi-square test for categorical variables were used to compare the characteristics and clinical data between the two groups. PRS was divided into quartiles to analyze the risk of myopia in different PRS quartiles. Odds ratios and 95% confidence intervals (CIs) were determined using a logistic regression analysis. The predictive accuracy of the PRS model was evaluated using the AUC adjusted for sex and age. The AUC of the PRS model adjusted for age and sex was determined using logistic regression analysis. All statistical analyses were conducted using SPSS version 22 (IBM Co., Ltd., Armonk, NY, USA) and R version 3.4.4 (R Core Team, 2018). A *p*-value less than 0.05 was considered statistically significant.

## 3. Results

### 3.1. Study Participants

In the discovery cohort with 10,156 participants, the mean ± SE of high myopia (mean age, 46.69 ± 14.79 years) and no myopia (mean age, 63.18 ± 13.50 years) participants was −9.77 ± 4.01 and 1.79 ± 2.72 D, respectively. The proportion of females was 60.8% in the high myopia group and 55.4% in the no myopia discovery group (Table 1). In the replication analysis, independent replication cohorts comprised individuals with no myopia and patients with mild, moderate, and high myopia aged >18 years. Additionally, patients aged 6–18 and ≤6 years with high or no myopia were included in our replication analysis. Details of the replication cohort characteristics are provided in Table 1 and Table 2.

### 3.2. PRS in the Replication Cohort with Participants Aged >18 Years

**Figure 3:**
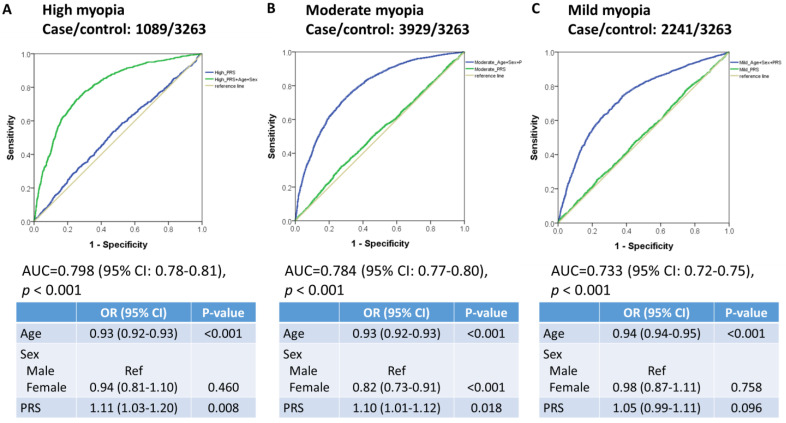
Receiver operating characteristic (ROC) curve for detecting (**A**) high, (**B**) moderate, and (**C**) mild myopia versus no myopia controls (SE > −1.0 D) with the PRS, age, and sex as predictors in participants aged >18 years; the AUC and 95% confidence interval (CI) correspond to the PRS, age, and sex models.

The PRS was evaluated in 10,522 participants, including those with high (SE < −6.0 D; n = 1089), moderate (−6.0 D < SE ≤ −3.0 D; n = 3929), mild (−3.0 D < SE ≤ −1.0 D; n = 2241), and no myopia (n = 3263). The PRS had an AUC of 0.53 (95% CI = 0.51–0.55, *p* = 0.003; Figure 3A), 0.52 (95% CI = 0.51–0.53, *p* = 0.002; Figure 3B), and 0.51 (95% CI = 0.50–0.53, *p* = 0.118; Figure 3C) for high, moderate, and mild myopia, respectively. Inclusion of age and sex in the PRS model increased the AUC to 0.80 (95% CI = 0.78–0.81, *p* < 0.001; Figure 3A), 0.78 (95% CI = 0.77–0.80, *p* < 0.001; Figure 3B), and 0.73 (95% CI = 0.72–0.75, *p* < 0.001; Figure 3C) for high, moderate, and mild myopia, respectively. Further, high PRS significantly increased the risk of high and moderate myopia (Figure 3A,B), and increasing age significantly decreased the risk of high, moderate, and mild myopia (Figure 3A–C). Furthermore, participants in the top PRS quartile had a 1.30-fold greater risk of high myopia (95% CI = 1.09–1.55, *p* = 0.003) compared with that in the participants in the bottom 75% PRS, as shown in Table 3.

### 3.3. PRS in the Replication Cohort with Participants Aged 7–18 and ≤6 Years

**Figure 4:**
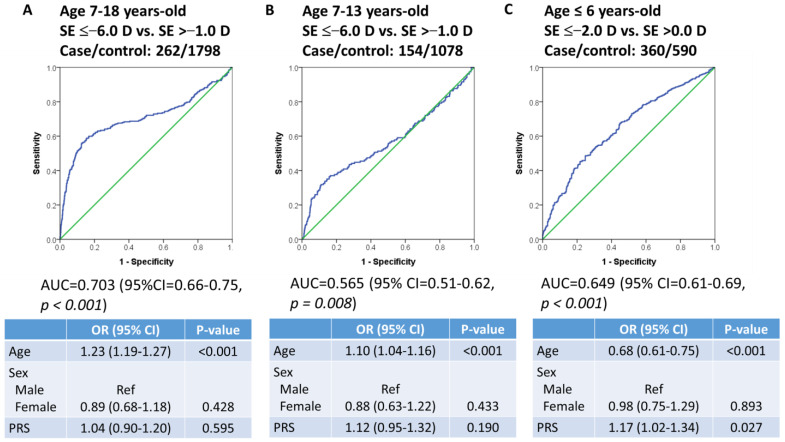
ROC curve for detecting high myopia in participants aged (**A**) 7–18, (**B**) 7–13, and (**C**) ≤6 years with the PRS, age, and sex as predictors (blue line); green line represented reference line. The AUC and 95% CI correspond to the PRS, age, and sex model.

We evaluated the PRS in independent cohorts with participants aged 7–18 years and ≤6 years. The PRS combined with age and sex had an AUC of 0.70 (95% CI = 0.66–0.75, *p* < 0.001; Figure 4A) and 0.57 (95% CI = 0.51–0.62, *p* = 0.008; Figure 4B) stratified for high myopia participants aged 7–18 or 7–13 years, respectively. Furthermore, increasing age significantly increased the risk of high myopia in participants aged 7–18 and 7–13 years (Figure 4A,B). Moreover, we evaluated the PRS in a cohort comprising children ≤ 6 years of age, including 360 children with SE ≤ −2.0 D and 590 children with SE > 0.0 D; the AUC of PRS, including age and sex, was 0.65 (95% CI = 0.61–0.69, *p* < 0.001; Figure 4C). A high PRS significantly increased the risk of high myopia, while increasing age significantly decreased the risk of high myopia in children ≤6 years of age (Figure 4C).

## 4. Discussion

In the current study, we generated a new PRS based on our GWAS outcomes in a Taiwanese population and integrated the results of previously published GWAS associated with myopia. Our findings indicate that the model, which includes age, sex, and PRS, exhibits a good predictive ability for high myopia, resulting in an improved AUC of 0.80, 0.78, and 0.73 for high, moderate, and mild myopia, respectively, in cohorts comprising individuals aged >18 years.

This genetic prediction method offers a substantial advantage over the cycloplegic refraction technique because it can be implemented in children at an early age, even before the onset of childhood hyperopia. However, considering the absence of any available prophylactic interventions to prevent myopia onset, apart from advising children to increase their outdoor activities and adopt a better reading posture, this advantage currently holds greater academic interest than practical recommendations for clinical practice. However, genetic prediction methods can still be useful for identifying high-risk groups requiring close follow-up and early intervention. Consequently, it is crucial to detect and treat myopia at an early stage to prevent its progression and associated complications.

GWASs have been widely applied over the past 20 years, with over 100 SNPs associated with myopia, but none have been proven to directly cause myopia. In the current study, our GWAS analysis revealed *SNTB1* as the gene most significantly associated with high myopia. This gene has been reported to be associated with moderate-to-high myopia in Han Chinese populations [30,31,32,33,34,35,36]. Previously, the most well-known studies conducted among Taiwanese and Japanese populations have identified significant associations of high myopia with the lumican gene SNPs rs3759223 and rs3741834 and the SNP rs577948 on chromosome 11q24.1 [37,38]. CHRM3 and TGFβ1 have been implicated in the pathogenesis of myopia in Taiwanese individuals, while a high-myopia GWAS of Asian datasets identified the SNP rs6885224 in the *CTNND2* gene on chromosome 5p15.2 as being associated with myopia [39,40,41]. Previous GWASs have established a solid foundation for PRS development, enabling clinicians to adopt a more personalized approach to myopia prevention and control. Using PRS with other predictive factors may help clinicians identify high-risk populations, particularly children, and guide interventions to reduce the risk of high myopia at a later age.

Currently, the largest PRS model for myopia prediction is derived from a meta-analysis of three GWASs comprising data from 711,984 participants, with a significant portion of the database obtained from the UK Biobank [14]. The resulting PRS exhibited a slightly inferior prediction performance (AUC of 0.67 [95% CI, 0.65–0.70] for any myopia, 0.75 (95% CI, 0.70–0.79) for moderate myopia, and 0.73 (95% CI, 0.66–0.80) for high myopia) compared to that of our own study. The incorporation of educational attainment into the PRS marginally improved the area under the ROC curve for myopia (0.674 vs. 0.668; *p* = 0.02) but demonstrated no significant improvements for moderate and high myopia. Given the difference in genetic backgrounds between European and Asian populations, PRS models developed using European data may not accurately predict myopia risk in Asian cohorts, resulting in lower predictive ability.

In our study, the PRS model had a relatively low AUC of 0.53, 0.52, and 0.51 for high, moderate, and mild myopia, respectively, in Taiwanese individuals aged >18 years. These results were not as favorable as those of a PRS model established in the Singapore cohort of risk factors for myopia (SCORM) study, which showed AUCs of 0.64 and 0.57 for high and moderate myopia, respectively, with a smaller sample size of 1004 individuals [16]. However, the Taiwanese replicate sample size was larger (10,522 individuals) than that of the SCORM study. Nevertheless, the inclusion of age and sex in the PRS model increased the AUCs to 0.80, 0.78, and 0.73 for high, moderate, and mild myopia, respectively, in the Taiwanese population. Similar results were observed in the SCORM study, with improvements in AUCs to 0.77 and 0.62 for high and moderate myopia, respectively, by including age, time spent outdoors, parental myopia, and PRS in the model [16]. In this study, females exhibited a slightly higher proportion of high myopia. Although changes in societal demands related to education and profession may contribute to an increased risk of myopia among females, women were predominant in multiple databases and across all age groups. This aligns with epidemiological studies in the general population, which have identified female sex as a risk factor for high myopia [42,43]. Recent studies reviewing high myopia prevalence from 1966 to 2019, based on the WHO definition (SE –5.0 D or less), reported prevalence rates ranging from 6.2% to 13.7% in females and 4.7% to 11.0% in males, with significant differences observed throughout the years [44]. Therefore, we incorporated sex into our PRS construction to improve the AUC.

The complex etiology of myopia involves genetic and environmental factors, as well as their interactions, which contribute to the clinical presentation and progression of myopia [45]. Consequently, considering other relevant factors in the prediction model could enhance its accuracy and clinical utility. The strength of our model is that age and sex are more feasible and objective variables than other variables such as time spent outdoors and parental myopia, which may have more subjective measurement or recall bias, as reported in the SCORM cohort study.

A recent study that monitored 1043 Chinese school-going children over three years concluded that *GJD2* rs524952, *KCNQ5* rs7744813, and *ZFHX1B* rs13382811 were significantly associated with fast myopic progression [30]. The same study indicated that *GJD2* and *KCNQ5* may affect myopia progression through axial elongation [30]. However, this study had certain limitations, including a small sample size, limited data points, and the selection of only one representative SNP for each gene. Our PRS model included these SNPs, which were selected based on their significant associations with myopic progression. However, further investigations on the association between PRS and myopic progression are needed in the future. Additionally, in contrast to common genetic loci with low effect sizes, environmental risk factors, such as proximity to work and time spent outdoors, have a strong impact on myopia etiology and progression [45,46].

According to a previous longitudinal study involving Chinese twin children, baseline refraction, age, and sex were sufficient to predict the risk of high myopia, but the addition of myopic PRS did not improve this model [47]. However, the PRS used in this study was not derived from an ancestrally matched population, potentially leading to incorrect estimates of the effect sizes of the included variants. Nonetheless, PRS may be useful in identifying individuals at high risk of pathological myopia, which results in irreversible vision loss because of progressive retinal atrophy, retinal detachment, or choroidal neovascularization [46,48]. This high-risk group may benefit from regular screening, counseling, and lifestyle changes, such as increased time spent outdoors, which may reduce disease progression [49].

The current study has certain limitations. First, the included replication groups were all Taiwanese populations, and there were no other study populations or ethnic groups to validate the SNPs identified in this study. This may lead to inaccurate myopia risk predictions and hinder the validation of PRS in different study populations or ethnic groups. In addition, environmental and lifestyle risk factors, such as near work and outdoor time, were not included and could not be combined with the PRS for myopia prediction. However, incorporating these variables as predictors of high myopia may introduce bias owing to incorrect recall or subjective measurements. Another limitation of this study using PRS to predict high myopia in children less than 6 years old is the limited number of children in this age group who meet the International Myopia Institute definition of high myopia (SE ≤ −6.00 D). However, we chose to define high myopia in children under 6 years of age as an SE refraction of less than −2.0 D. This decision was based on findings from previous longitudinal studies on premyopic children [19], which suggested that the average SE progressed to less than −6.0 D by the age of 12 if left untreated. Another limitation was the inclusion of subjects under 18 years of age, which added complexity and heterogeneity to the study population. However, these participants were included because we aimed to predict myopia at younger ages. Further, the criteria for determining whether the subjects underwent anti-myopic treatment were not clearly defined. However, according to our healthcare policy, all preschoolers undergo mandatory visual acuity screening at the age of 4 years, with annual examinations mandated for school-aged children across Taiwan. Any child who failed to meet the vision screening standards was referred to a hospital with ophthalmologists for a thorough eye examination. Therefore, we can assume that most children with myopia were successfully treated. Furthermore, this study was cross-sectional and not longitudinal, and ocular predictors such as myopia onset age or severity of myopia in childhood were not available. Future prospective longitudinal studies incorporating more comprehensive variables are thus necessary to establish a more accurate PRS model.

## 5. Conclusions

Overall, the PRS, age, and sex improved the prediction of high myopia risk in individuals aged >18 years. Individuals in the top quartile of the PRS had an increased risk of developing high myopia. However, further predictive studies with genetic loci from GWAS studies on myopia in East Asians and detailed analyses of ocular and lifestyle factors may be required to increase the predictive performance to an acceptable level for use in clinical applications.

## Figures and Tables

**Table 1:** Characteristics of participants aged >18 years in the discovery and replication cohorts.

	Discovery Cohort (n = 10,156)		Replication Cohort (n = 10,522)			
	No Myopia ^1^	High Myopia ^2^	No Myopia ^1^	High Myopia ^2^	Moderate Myopia ^3^	Mild Myopia ^4^
Sample size	7615	2541	3263	1089	3929	2241
Age (years, mean ± SD)	63.18 ± 13.50	46.69 ± 14.79 **	63.12 ± 13.43	47.10 ± 14.36 **	47.77 ± 14.78 **	51.14 ± 14.96 **
Sex						
Male (%)	3396 (44.6)	997 (39.2) **	1398 (42.8)	443 (40.7)	1681 (42.8)	905 (40.4)
Female (%)	4219 (55.4)	1544 (60.8)	1865 (57.2)	646 (59.3)	2248 (57.2)	1336 (59.6)
SE in the worse eye (D, mean ± SD)	1.79 ± 2.72	(−9.77 ± 4.01) **	1.76 ± 2.72	(−9.70 ± 3.94) **	(−4.34 ± 0.85) **	(−2.03 ± 0.51) **

^1^ SE > −1.0 D, ^2^ SE ≤ −6.0 D, ^3^ SE > 0.0 D, ^4^ SE ≤ −2.0 D. ** *p* < 0.001 compared with no myopia group.

**Table 2:** Characteristics of participants aged ≤18 years in the replication cohort.

	Replication Cohort					
	7–18		7–13		≤6	
	No Myopia ^1^	High Myopia ^2^	No Myopia ^1^	High Myopia ^2^	No Myopia ^3^	High Myopia ^4^
Sample size	1798	262	1678	154	590	360
Age (years, mean ± SD)	6.14 ± 3.67	10.31 ± 5.61 **	5.49 ± 2.82	6.39 ± 3.87 *	3.82 ± 1.29 **	3.14 ± 1.36 **
Sex						
Male (%)	831 (46.2)	129 (49.2)	778 (46.4)	77 (50.0)	266 (45.1)	169 (46.9)
Female (%)	967 (53.8)	133 (50.8)	900 (53.6)	77 (50.0)	324 (54.9)	191 (53.1)
SE in the worse eye (D, mean ± SD)	0.89 ± 2.07	(−9.28 ± 3.66) **	0.89 ± 2.08	(−9.61 ± 3.90) **	1.68 ± 2.27 **	(−4.98 ± 3.46) **

^1^ SE > −1.0 D, ^2^ SE ≤ −6.0 D, ^3^ −6.0 D < SE ≤ −3.0 D, ^4^ −3.0 D < SE ≤ −1.0 D. * *p* = 0.005, ** *p* < 0.001 compared with no myopia group.

**Table 3:** Odds ratios (ORs) of high, moderate, and mild myopia for participants aged >18 years in the top PRS quartile.

Risk	Reference	Degree of Myopia	OR (95% CI)	*p*-Value
Top 25%	Remaining 75%	High	1.30 (1.09–1.55)	0.003
		Moderate	1.23 (1.09–1.39)	0.001
		Mild	1.07 (0.94–1.23)	0.298

## Data Availability

The data presented in this study are available on request from the corresponding author. The data are not publicly available due to participant privacy.

## Supplementary Materials

The following supporting information can be downloaded at: https://www.mdpi.com/article/10.3390/biomedicines12071619/s1, Table S1: 34 GWAS Catalog studies were used for PRS construction; Table S2: 149 SNPs were used to generate the PRS model.
