# Supplementary material for: Developing a Polygenic Risk Score with Age and Sex to Identify High-Risk Myopia in Taiwan
Source: Biomedicines. 2024 Jul 20;12(7):1619. doi: 10.3390/biomedicines12071619 (PMC11274619; doi:10.3390/biomedicines12071619)
Supplement: Supplementary file 1 [file biomedicines-12-01619-s001.zip › Supplementary Table S1 (34 GWAS catalog studies).pdf]

**Supplementary**

**Table S1. 34 GWAS Catalog studies were used for PRS construction**

| GWAS catalog accession ID | Pubmed ID | Discovery Sample Ancestry | Replication Sample Ancestry |
|---|---|---|---|
| GCST000491 | 19779542 | 1231 East Asian | 1510 East Asian |
| GCST000878 | 21095009 | 980 East Asian | 3087 East Asian |
| GCST001044 | 21505071 | 437 East Asian | 12962 East Asian |
| GCST001088 | 21640322 | 1088 East Asian | 8445 East Asian |
| GCST001561 | 22685421 | 2155 South East Asian, 2789 East Asian | |
| GCST001712 | 23049088 | 1251 European | |
| GCST001881 | 23406873 | 1625 East Asian | 5811 East Asian |
| GCST002117 | 23933737 | 5030 East Asian | 4800 East Asian |
| GCST002615 | 25233373 | 15619 European | 3919 NR, 4581 European |
| GCST003997 | 27182965 | 191843 European | |
| GCST009962 | 31816047 | 50372 European | |
| GCST010994 | 32428537 | 3218 East Asian | 5969 East Asian, 4858 South East Asian, East Asian |
| GCST012379 | 34241624 | 527 African American or Afro-Caribbean | |
| GCST012400 | 33830181 | 22025 European | 2469 European, 739 Asian unspecified |
| GCST012402 | 33830181 | 32613 European | |
| GCST012403 | 33830181 | 24580 European | |
| GCST90044218 | 34737426 | 36623 European | |
| GCST90044219 | 34737426 | 36623 European | |
| GCST90044220 | 34737426 | 36623 European | |
| GCST90044326 | 34737426 | 455654 European | |
| GCST90078393 | 34662886 | 94332 European | |
| GCST90078394 | 34662886 | 94332 European | |
| GCST90079388 | 34662886 | 51135 European | |
| GCST90079389 | 34662886 | 51135 European | |
| GCST90079390 | 34662886 | 51135 European | |
| GCST90079916 | 34662886 | 387930 European | |
| GCST90082379 | 34662886 | 94332 European | |
| GCST90082380 | 34662886 | 94332 European | |
| GCST90083374 | 34662886 | 51135 European | |
| GCST90083375 | 34662886 | 51135 European | |
| GCST90083376 | 34662886 | 51135 European | |
| GCST90083902 | 34662886 | 387930 European | |
| GCST90095188 | 35116054 | 801 East Asian | 55745 East Asian |
| GCST90134549 | 35841873 | 64268 European | |
