# Supplementary material for: Developing a Polygenic Risk Score with Age and Sex to Identify High-Risk Myopia in Taiwan
Source: Biomedicines. 2024 Jul 20;12(7):1619. doi: 10.3390/biomedicines12071619 (PMC11274619; doi:10.3390/biomedicines12071619)
Supplement: Supplementary file 1 [file biomedicines-12-01619-s001.zip › Supplementary Table S2 (149 SNPs used to generate the PRS model).pdf]

Supplementary

Table 2. 149 SNPs were used to generate the PRS model

chr	bp	SNP rs number	Reference allele	Odd ratios	P value
1	11944305	rs9730417	A	1.21686	7.54E-05
1	12639168	rs3128461	G	1.14774	9.45E-05
1	58673489	rs12042368	A	1.29692	4.79E-05
1	63645691	rs57849430	A	0.80332	6.26E-05
1	69148593	rs77091935	C	1.18204	1.25E-05
1	70138465	rs12025135	G	0.84310	5.25E-05
1	80321346	rs188186208	C	1.52742	7.64E-05
1	101910587	rs61784369	A	1.14555	8.63E-05
1	107842216	rs117354873	A	1.45404	4.82E-05
1	116372354	rs36097042	T	0.77609	2.74E-05
1	168828841	rs200575	G	0.81460	4.29E-05
1	169298423	rs10919141	C	0.85543	9.75E-05
1	201642166	rs576570362	C	0.47211	8.81E-05
1	208060807	rs2745951	G	0.87428	7.49E-05
1	226830381	rs1151803	G	0.84620	1.41E-05
1	227567143	rs144930899	T	1.75478	3.66E-05
1	246838747	rs9426261	C	0.87472	7.11E-05
2	7526194	rs571887173	C	1.77147	6.83E-05
2	9876728	rs4668641	A	1.14973	7.39E-05
2	16492636	rs4832489	C	0.84259	8.01E-05
2	18351537	rs2011027	G	1.14518	6.68E-05
2	34342896	rs59495126	T	0.77181	5.92E-06
2	79637894	rs13022854	T	0.85058	2.12E-05
2	129559762	rs10185924	G	1.15428	1.13E-05
2	130323041	rs58215584	C	1.15425	1.30E-05
2	139836023	rs963629	T	1.15014	8.91E-05
2	143709091	rs12997823	G	0.87535	5.83E-05
2	144677535	rs56266000	C	1.13969	7.26E-05
2	149674070	rs117312764	A	1.90454	6.44E-06
2	156813021	rs117321465	G	1.59711	1.32E-05
2	156933377	rs12471552	A	1.14160	4.96E-05
2	224110366	rs1517653	C	0.84065	1.90E-05
2	233385396	rs1550094	A	1.25112	4.93E-05
3	10020524	rs55850027	A	1.25676	7.03E-05

3	10308326	rs1642974	G	0.84743	7.66E-06
3	38210029	rs34498353	G	1.30651	5.13E-05
3	56826791	rs17216768	C	0.87463	6.32E-05
3	61651299	rs35289930	A	1.20627	4.29E-05
3	76418451	rs7649237	G	0.86491	3.13E-05
3	111348019	rs4682294	G	0.64827	5.82E-05
3	114713823	rs4682167	C	0.85422	3.67E-05
3	124699509	rs150554398	T	0.70294	7.16E-05
3	150636224	rs936189	T	1.15036	4.45E-05
4	20625689	rs1457916	T	1.32462	4.78E-05
4	38702973	rs10026640	C	1.20309	8.68E-06
4	106610368	rs116957480	A	0.72672	7.23E-05
4	129532623	rs1216419	A	0.85592	5.82E-05
4	145363247	rs1602238	T	1.19962	5.18E-05
4	170877171	rs75042183	C	0.80552	6.86E-05
5	525945	rs185641736	A	1.60235	5.55E-05
5	2131123	rs12521827	T	1.20254	1.77E-05
5	3240272	rs620704	A	0.86798	7.78E-05
5	9965794	rs6867995	C	1.14923	1.93E-05
5	20358490	rs59032817	G	0.82924	5.48E-05
5	29892677	rs28418554	C	0.87194	6.20E-05
5	112206001	rs149142888	C	0.57080	2.34E-05
5	119639313	rs191358705	C	1.41132	8.98E-05
5	142507171	rs3797074	C	1.18376	4.30E-05
5	169365383	rs77585674	G	0.81682	7.64E-05
6	18030079	rs9383343	A	1.21267	7.39E-05
6	109804311	rs13437139	A	1.71041	9.60E-05
6	119640549	rs2357376	C	1.16079	4.89E-05
6	119756216	rs9374795	A	1.43300	8.05E-05
6	146808416	rs76382764	A	1.37693	2.12E-05
6	170462892	rs9460056	T	0.84486	8.08E-05
6	170696385	rs6934743	G	1.21033	2.74E-05
7	20351465	rs2286695	A	0.86399	7.63E-06
7	24178848	rs198724	G	0.85472	5.00E-05
7	47859552	rs80279956	G	1.32349	6.63E-05
7	77818659	rs11771183	C	1.15110	5.71E-05
7	80779426	rs327670	G	0.86559	3.27E-05
7	95072616	rs10953147	G	0.85135	4.31E-05

7	109811345	rs2396210	C	1.16201	1.80E-05
7	112281687	rs9690753	G	0.83749	8.32E-05
7	120484682	rs147509162	T	1.36031	8.39E-05
7	130888388	rs17165483	G	1.15511	1.35E-05
7	158523837	rs17837836	T	0.64436	3.27E-05
7	158898659	rs55950988	T	0.82726	4.98E-05
7	158994960	rs144280192	T	1.23963	1.66E-05
8	23927602	rs184363924	C	0.40173	2.57E-05
8	103844082	rs117434728	C	1.29730	9.02E-05
8	121610438	rs6469937	A	0.81692	8.63E-08
8	136859008	rs10113544	A	1.16177	6.82E-05
9	7843394	rs3847268	A	0.77436	3.04E-05
9	74338896	rs79637440	C	0.76072	6.38E-05
9	78970189	rs60612492	C	1.80007	3.55E-05
9	85763400	rs4621894	C	0.78295	4.99E-05
9	90802614	rs17080774	T	0.76439	8.69E-05
9	104461050	rs77325969	G	1.83739	1.22E-05
9	127808611	rs28481397	T	1.14871	3.07E-05
9	138447810	rs7019967	G	1.26023	2.39E-05
10	7008503	rs72638724	G	0.87397	5.46E-05
10	12013175	rs76521633	A	1.47234	2.65E-05
10	12464305	rs10795953	C	1.14240	6.34E-05
10	129747299	rs143359234	T	1.66800	2.49E-05
10	135386067	rs71484087	A	0.84766	9.33E-05
11	61643343	rs188187545	A	0.47829	5.76E-05
11	84219732	rs57098434	T	0.71556	6.58E-05
11	114070242	rs3782005	A	1.15243	1.96E-05
12	12074809	rs75253437	G	0.69980	6.12E-05
12	12566517	rs531688710	G	1.69429	6.35E-05
12	20967682	rs2417946	A	0.84131	9.26E-05
12	68555011	rs2069705	A	0.84279	6.88E-06
12	116764284	rs143783238	T	1.48385	2.30E-06
12	127271615	rs9634139	T	1.36693	3.59E-05
12	130897844	rs10848097	C	0.84669	7.07E-05
13	37493676	rs478811	A	0.84753	3.38E-05
13	43418003	rs9594821	T	0.87113	8.32E-05
13	51867290	rs2153512	T	1.13806	8.85E-05
13	75825210	rs1864726	C	1.31711	8.30E-05

13	96897856	rs9562057	T	1.16274	1.55E-05
13	101834378	rs650159	A	1.14967	9.59E-05
13	102425912	rs9518532	G	1.18956	6.18E-05
14	46363996	rs17647857	G	1.15599	7.51E-05
14	65402508	rs370120147	G	1.28931	4.07E-05
15	33011127	rs7168877	A	1.16074	4.41E-05
15	33265838	rs2930133	C	0.84665	9.09E-05
15	51634255	rs2446421	G	0.87672	8.07E-05
15	78718899	rs2869045	C	0.84730	1.50E-05
15	79378775	rs13380109	A	1.14398	3.50E-05
15	79987715	rs16971249	C	0.83735	1.90E-05
15	87129176	rs8042696	T	1.17958	4.01E-06
16	13217126	rs78628301	T	0.84865	4.21E-05
16	62533454	rs149868768	C	1.40906	2.55E-05
16	79706540	rs7202770	G	1.13928	6.20E-05
16	86846495	rs11644263	T	0.87503	5.09E-05
16	89649675	rs455868	A	0.86526	1.18E-05
17	85192	rs577448043	A	0.60581	7.37E-05
17	707164	rs882931	T	1.24277	9.30E-05
17	10796661	rs202591	T	1.14110	5.34E-05
17	37432616	rs75625292	G	0.73588	7.83E-05
17	64976121	rs3785594	T	0.85919	6.68E-05
17	66483281	rs7350964	A	0.83158	1.14E-05
18	38416382	rs79032281	T	0.64369	3.84E-05
18	49902879	rs34018179	G	0.80519	9.08E-05
18	52440040	rs140403789	C	1.44519	5.85E-05
18	72073738	rs73470570	C	0.82737	7.46E-05
18	72861714	rs2439252	A	1.15393	5.41E-05
18	76337293	rs117258879	T	1.36996	5.37E-06
19	1509035	rs112207132	A	0.87261	4.52E-05
19	58386038	rs145849148	T	1.37407	4.55E-05
20	605262	rs73894811	T	1.57126	5.72E-05
20	25236377	rs117974689	C	0.48077	6.21E-05
20	47098774	rs79588463	A	0.67383	5.55E-05
20	59626495	rs62199105	G	0.85689	2.29E-05
21	36026192	rs117444979	A	0.75014	7.74E-05
21	41366520	rs9977570	A	0.81853	9.40E-05
22	30371350	rs9614123	G	1.17465	3.40E-05

22	45793574	rs117372954	T	1.53151	8.99E-06
